# Severity of Cognitive Impairment and Mortality After Hip Fracture: Implications for Risk Stratification and Care Allocation

**DOI:** 10.3390/jcm15124506

**Published:** 2026-06-10

**Authors:** Sara Silvaieh, Arastoo Nia, Stephan Heisinger, Domenik Popp

**Affiliations:** 1 Department of Neurology, Medical University of Vienna, Waehringer Guertel 18-20, 1090 Vienna, Austria; 2 Comprehensive Center for Clinical Neurosciences and Mental Health, Medical University of Vienna, Waehringer Guertel 18-20, 1090 Vienna, Austria; 3 Clinical Division of Traumatology, Department of Orthopedics and Trauma Surgery, Medical University of Vienna, Waehringer Guertel 18-20, 1090 Vienna, Austriadomenik.popp@meduniwien.ac.at (D.P.)

**Keywords:** hip fracture, risk prediction, mortality, cognitive impairment, Nottingham Hip Fracture Score, Almelo Hip Fracture Score, American College of Surgeons National Surgical Quality Improvement Program

## Abstract

**Background/Objectives**: Proximal femur fracture (PFF) is associated with high mortality in older adults. Cognitive impairment is common in this population and linked to adverse outcomes, but differences in mortality between mild cognitive impairment (MCI) and dementia and the performance of prediction models in these subgroups remain unclear. **Methods**: A retrospective cohort study included 1579 patients aged ≥ 60 years undergoing surgery for PFF between 2017 and 2019. Patients were stratified into no, MCI and dementia. The primary outcome was all-cause mortality at 2 years, with additional analyses at predefined timepoints. Associations were assessed using Kaplan–Meier analysis, Cox regression, and timepoint-specific logistic regression. The performance of three prediction models (NHFS, AHFS, ACS-NSQIP) was evaluated using discrimination (AUC) and calibration metrics. **Results**: Cognitive impairment was present in 469 patients (29.3%), including 307 (19.2%) with mild and 162 (10.1%) with dementia. Mortality increased substantially with cognitive impairment, reaching 24.0% in patients without impairment, 43.7% with mild impairment, and 62.4% with dementia at 2 years. Both mild and severe impairment were independently associated with increased mortality (adjusted hazard ratios 1.48 and 1.95, respectively). Dementia was associated with early mortality, while MCI showed a delayed effect becoming significant from 6 to 12 months onward. Survival differed significantly between patients with and without impairment, but not between MCI and dementia groups. All three risk scores showed moderate and comparable discrimination (AUC ~0.71–0.75). ACS-NSQIP demonstrated the best calibration, while NHFS and AHFS tended to overestimate risk. **Conclusions**: Cognitive impairment is a strong independent predictor of mortality after PFF. Cognitive impairment was associated with increased mortality after proximal femur fracture. Associations differed across follow-up timepoints, although survival differences between patients with mild cognitive impairment and dementia were not statistically significant in unadjusted Kaplan–Meier analyses. These findings support the clinical relevance of cognitive impairment as a marker of vulnerability in this population.

## 1. Introduction

Proximal femur fracture is a major cause of morbidity and mortality in older adults and represents a growing challenge for healthcare systems worldwide [1]. Despite improvements in surgical techniques and perioperative care, mortality remains high, particularly in the first months after injury, and continues to increase over longer follow-up periods [2]. Outcomes are not determined by the fracture alone but reflect a close interaction of age, frailty, comorbidity, and functional reserve. Early identification of patients at increased risk is therefore essential to guide clinical decision-making and optimise postoperative care pathways [3].

Cognitive impairment is highly prevalent among patients with hip fracture and is consistently associated with worse clinical outcomes [4]. It is linked to reduced mobility, increased dependence, higher rates of institutionalisation, and a greater burden of systemic disease. In the perioperative setting, cognitive impairment also predisposes to complications such as delirium, impaired rehabilitation, and treatment non-adherence, all of which may contribute to excess mortality [5]. However, cognitive impairment is often considered as a single entity, although it encompasses a broad clinical spectrum. The extent to which mortality differs between MCI and dementia after proximal femur fracture remains unclear. Patients with dementia may experience greater early postoperative vulnerability due to impaired physiological reserve, dependence, and reduced rehabilitation potential, whereas patients with MCI may demonstrate a more delayed trajectory of functional decline.

Understanding whether mortality differs according to cognitive impairment severity may improve perioperative risk stratification and help identify vulnerable patient subgroups requiring closer clinical attention after hip fracture. Patients at highest risk may benefit from intensified geriatric co-management, closer monitoring, earlier and more structured physiotherapy, and shorter follow-up intervals. Conversely, recognising elevated risk even in mild cognitive impairment (MCI) may support broader implementation of targeted interventions in this subgroup.

Several risk prediction models have been developed to estimate mortality after hip fracture, including the Nottingham Hip Fracture Score (NHFS) [6], the Almelo Hip Fracture Score (AHFS) [7], and the American College of Surgeons National Surgical Quality Improvement Program risk calculator (ACS-NSQIP) [7]. These tools are widely used in clinical practice, yet their performance in patients with cognitive impairment has not been fully established. Because cognitive impairment is associated with multiple interacting risk factors, the accuracy of these models may differ in this subgroup. Reliable prediction in cognitively impaired patients is particularly important, as these individuals are among those most likely to benefit from targeted allocation of healthcare resources.

The aim of this study was to quantify the association between cognitive impairment and mortality after proximal femur fracture, with separate evaluation of MCI and dementia across short-term and long-term follow-up. Because cognitive impairment is commonly incorporated as a dichotomous variable within existing mortality prediction tools, differences across the spectrum of cognitive impairment severity may not be adequately reflected. In addition, the performance of three commonly used mortality prediction models, NHFS, AHFS, and ACS-NSQIP, was assessed in this population to determine their ability to discriminate and accurately predict outcomes. The objective was to identify whether differences in cognitive impairment severity translate into clinically meaningful differences in risk and whether existing prediction tools can support targeted allocation of postoperative resources and follow-up intensity.

## 2. Methods

### 2.1. Study Design and Population

This retrospective observational cohort study was conducted at the Clinical Division of Traumatology, Department of Orthopaedics and Trauma Surgery, Medical University of Vienna, Vienna, Austria, a level 1 trauma center. All consecutive patients aged 60 years or older who were admitted with a proximal femur fracture and underwent operative treatment during the predefined study period (January 2017 to December 2019) were screened for eligibility.

Eligible fractures included femoral neck, pertrochanteric, and subtrochanteric fractures. Patients managed non-operatively, those with pathological or periprosthetic fractures, patients with polytrauma, and cases with missing baseline information precluding cognitive classification or calculation of mortality prediction tools were excluded.

The study followed the STROBE recommendations for observational studies. Ethical approval was obtained from the local ethics committee (EK 1517/2020). The requirement for individual informed consent was waived due to the retrospective design.

### 2.2. Data Sources and Baseline Variables

Data were extracted from the institutional electronic medical record system and analysed in pseudonymised form. Collected baseline variables included age, sex, body mass index, American Society of Anesthesiologists (ASA) class, institutional residence, fracture type, surgical treatment, and comorbidities. Comorbidities comprised cardiac disease, chronic obstructive pulmonary disease, renal disease, and diabetes mellitus.

Fractures were categorized as femoral neck (medial), femoral neck (lateral), pertrochanteric, or subtrochanteric. Surgical treatment was categorized as intramedullary fixation, hemiarthroplasty, total hip arthroplasty, dynamic hip screw, or cannulated screw fixation.

### 2.3. Cognitive Impairment

Baseline cognitive status was classified into three categories: no cognitive impairment, mild cognitive impairment, and dementia. Classification was based on a structured review of the medical record at the time of admission, integrating (i) previously documented diagnoses (e.g., dementia or mild cognitive impairment), (ii) results from cognitive screening instruments, including the Mini-Mental State Examination (MMSE) where available, and (iii) contemporaneous preoperative clinical documentation of cognitive function.

When a formal diagnosis of dementia or mild cognitive impairment was documented, the documented diagnosis was retained. In the absence of a formal diagnosis, classification was based on a structured review of the medical record, integrating previously documented diagnoses, available MMSE results, and contemporaneous clinical documentation of cognitive function.

While a score < 24 is commonly used to indicate cognitive impairment, further categorization into no impairment (≥27), mild impairment (24–26), and dementia (≤23) was applied for analytical purposes. This approach was intended for epidemiological stratification within a retrospective dataset and does not replace a formal neuropsychological diagnosis.

As MMSE was not available in all patients, classification was based on all available clinical information. Only preoperative cognitive assessments and documentation available at the time of admission were considered for cognitive classification; postoperative delirium was therefore not incorporated into the classification process.

Patients without evidence of cognitive impairment served as the reference group.

### 2.4. Surgical Management

Patients were managed according to the institutional hip-fracture pathway. Treatment decisions were based on fracture morphology, biological age, pre-fracture mobility, and overall medical condition. Femoral neck fractures were treated with internal fixation, hemiarthroplasty, or total hip arthroplasty as clinically indicated. Extracapsular fractures were treated predominantly with intramedullary fixation or, in selected stable patterns, dynamic hip screw constructs. Perioperative optimization followed a multidisciplinary protocol involving trauma surgeons, anesthesiologists, and internal medicine specialists.

### 2.5. Outcomes and Prediction Models

The primary outcome was all-cause mortality within 2 years after surgery. Mortality was additionally assessed at predefined timepoints of 30 days, 3 months, 6 months, 1 year, and 18 months. Time-to-event was defined as the interval between date of surgery and date of death or censoring at last available follow-up.

Three commonly used perioperative mortality prediction tools were evaluated:

The Nottingham Hip Fracture Score (NHFS) is a hip fracture–specific model derived from a large UK cohort that incorporates age, sex, admission hemoglobin, cognitive status, institutional residence, comorbidity burden, and malignancy to estimate 30-day mortality.

The American College of Surgeons National Surgical Quality Improvement Program (ACS-NSQIP) risk calculator is a general surgical model based on 21 variables, including demographics, functional status, comorbidities, and procedural factors, to estimate postoperative mortality and complications. Although not specific to hip fractures, it has been applied in orthopaedic populations.

The Almelo Hip Fracture Score (AHFS) extends the NHFS by incorporating ASA class and Parker Mobility Score, thereby integrating physiological reserve and pre-fracture function, and stratifies patients into risk categories for early mortality.

Scores were calculated using the earliest available preoperative clinical and laboratory data. Cases with missing baseline information precluding cognitive classification or nutritional score calculation were excluded from analysis.

### 2.6. Statistical Analysis

Continuous variables are presented as mean (SD) or median (range), as appropriate. Categorical variables are presented as *n* (%). Cumulative mortality proportions with 95% confidence intervals were calculated for each predefined timepoint. Kaplan–Meier survival curves were generated to assess survival according to cognitive impairment status, and differences between groups were evaluated using the log-rank test. Pairwise comparisons were adjusted using the Holm–Bonferroni method.

Univariable and multivariable Cox proportional hazards models were used to assess the association between baseline cognitive impairment and 2-year mortality. Hazard ratios (HRs) with 95% confidence intervals were calculated. The multivariable model was adjusted for age, sex, ASA class, institutional residence, and fracture type.

Timepoint-specific associations were evaluated using univariable and multivariable logistic regression analyses for mortality at 30 days, 3 months, 6 months, 1 year, 18 months, and 2 years. Odds ratios (ORs) with 95% confidence intervals were reported. Multivariable models were adjusted for the same covariates as the Cox model. *p* values were corrected for multiple comparisons using the Holm–Bonferroni method.

Discriminatory performance of NHFS, AHFS, and ACS-NSQIP was assessed using receiver operating characteristic analysis with calculation of the area under the curve (AUC) and corresponding 95% confidence intervals at each timepoint. Pairwise comparisons of AUCs were performed using DeLong’s test with Holm correction.

Calibration was assessed using calibration intercepts, calibration slopes, and Brier scores at each timepoint. An intercept of 0 and slope of 1 were considered indicative of ideal calibration, and lower Brier scores indicated better predictive accuracy.

All tests were two-sided, and a *p* value of less than 0.05 was considered statistically significant. Statistical analyses were performed using SPSS software for Mac (version 21, IBM, SPSS).

## 3. Results

The final cohort consisted of 1579 patients; 469 (29.3%) had cognitive impairment at baseline, including 307 (19.2%) with MCI and 162 (10.1%) with dementia. Patients with cognitive impairment represented a distinctly more vulnerable subgroup, characterised by advanced age (mean 83.2 years; SD 9.4) in severe impairment vs. 79.9 years (SD 9.1 without impairment) and a markedly higher burden of systemic disease.

The distribution of ASA class shifted towards more severe systemic disease with increasing cognitive impairment, with a higher proportion of patients classified as ASA III and IV in the impaired groups. Institutionalisation was markedly more common among cognitively impaired patients, affecting 79 (47.0%) of those with dementia compared with 155 (14.0%) without impairment, indicating substantially greater baseline dependency (Table 1).

Fracture patterns differed modestly between groups, with pertrochanteric fractures more frequent in patients with cognitive impairment. Correspondingly, intramedullary fixation was more commonly performed in these patients. Comorbid conditions, including cardiac disease, chronic obstructive pulmonary disease, renal disease, and diabetes, were consistently more prevalent in patients with cognitive impairment, reflecting a more vulnerable clinical profile.

Body mass index decreased slightly with increasing cognitive impairment, although the magnitude of this difference was small.

Mortality increased substantially with cognitive impairment across all timepoints (Table 2). At 30 days, mortality was 2.5% (28/1110) in patients without impairment, compared with 11.1% (34/307) and 14.2% (23/162) in those with MCI and dementia, respectively. This difference widened progressively over time, reaching 24.0% (266/1110), 43.7% (134/307), and 62.4% (101/162) at 2 years.

Mortality was consistently higher among cognitively impaired patients across all evaluated timepoints compared with cognitively unimpaired patients. Mortality estimates were numerically higher in dementia compared with MCI throughout follow-up, although subgroup differences were less pronounced than the overall distinction between cognitively impaired and unimpaired patients (Table 2). Overall, these findings demonstrate a strong and persistent association between cognitive impairment and mortality after proximal femur fractures.

**Figure 1:**
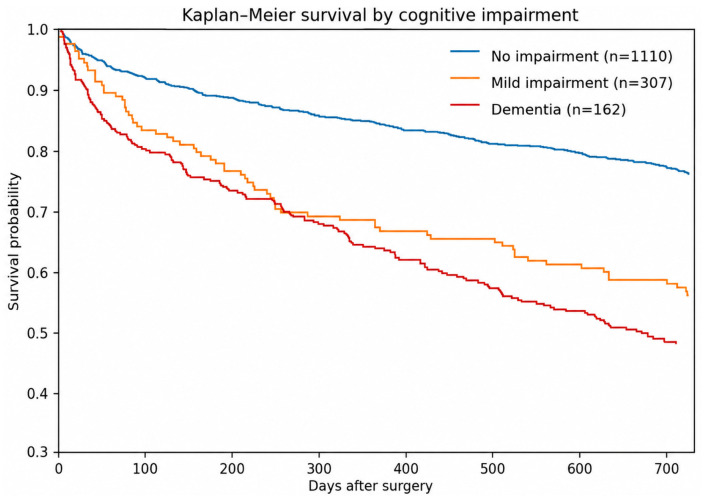
Kaplan–Meier survival curves stratified by cognitive impairment following PFF surgery. Kaplan–Meier survival curves for 2-year mortality stratified by cognitive impairment severity. Patients with mild and severe cognitive impairment showed significantly reduced survival compared with patients without impairment (overall log-rank *p* < 0.001).

Kaplan–Meier analysis demonstrated a clear separation of survival curves according to cognitive impairment severity, with progressively poorer survival observed in patients with MCI and dementia compared with those without impairment (Figure 1). The overall log-rank test indicated a highly significant difference between groups (χ^2^ = 112.14, *p* < 0.001).

In pairwise comparisons, survival differed significantly between patients without impairment and those with MCI (χ^2^ = 32.96, *p* < 0.001; Holm-adjusted *p* < 0.001) and between patients without impairment and those with dementia (χ^2^ = 103.62, *p* < 0.001; Holm-adjusted *p* < 0.001). In contrast, there was no statistically significant difference between patients with MCI and dementia (χ^2^ = 2.41, *p* = 0.120; Holm-adjusted *p* = 0.120). This indicates that the main survival difference is between patients with any cognitive impairment and those without impairment, whereas the difference between MCI and dementia was not statistically significant. These findings suggest that the principal survival difference was observed between cognitively impaired and cognitively unimpaired patients overall, whereas subgroup differences between MCI and dementia were less pronounced in unadjusted survival analysis.

In contrast, multivariable regression analyses accounting for baseline clinical characteristics demonstrated a graded association with mortality.

Overall, the principal survival difference was observed between cognitively impaired and cognitively unimpaired patients, whereas subgroup differences between MCI and dementia were less pronounced in unadjusted survival analysis.

In Cox regression analysis, baseline cognitive impairment was strongly associated with increased 2-year mortality (Table 3). In univariable analysis, both MCI and dementia impairment were associated with more than a twofold increased hazard of death compared with patients without impairment (mild: HR 2.12, 95% CI 1.63–2.75; severe: HR 2.63, 95% CI 2.16–3.19; both *p* < 0.001).

After adjustment for age, sex, ASA class, institutional residence, and fracture type, associations between cognitive impairment and mortality remained statistically significant, although attenuated. Mild cognitive impairment was associated with a 48% increased hazard of death (HR 1.48, 95% CI 1.06–2.08, *p* = 0.022), while dementia was associated with a nearly twofold increased hazard (HR 1.95, 95% CI 1.54–2.49, *p* < 0.001). This attenuation indicates partial confounding by baseline characteristics; however, the association between cognitive impairment and mortality persisted after adjustment for major baseline covariates.

Among covariates, increasing age, male sex, higher ASA class, and institutional residence were all independently associated with mortality (all *p* ≤ 0.027), whereas fracture type was not. Notably, the magnitude of effect for dementia was comparable to that of established clinical risk factors, supporting its clinical relevance within this population (Table 3).

Timepoint-specific logistic regression analyses demonstrated a consistent and time-dependent association between cognitive impairment and mortality (Table 4).

In univariable analyses, dementia was associated with increased mortality at all timepoints, including early mortality at 30 days (OR 3.54, 95% CI 1.71–7.34; Holm-adjusted *p* = 0.002), whereas MCI was not associated with early mortality. From 3 months onward, both MCI and dementia were associated with increased mortality, with progressively increasing effect sizes over time.

After multivariable adjustment and correction for multiple testing, differences across follow-up periods were observed. Dementia remained a robust and consistent predictor of mortality from 3 months through 2 years, with statistically significant associations across all timepoints (all Holm-adjusted *p* ≤ 0.010). In contrast, mild cognitive impairment showed less consistent associations at earlier follow-up timepoints, losing statistical significance at early timepoints but remaining independently associated with mortality at 1 year and beyond.

At 2 years, mild cognitive impairment was associated with an adjusted odds ratio (OR) of 2.42 (95% CI 1.44–4.05; Holm-adjusted *p* = 0.007), while dementia was associated with a substantially higher risk (OR 4.79, 95% CI 3.06–7.51; Holm-adjusted *p* < 0.001).

These findings suggest that cognitive impairment was associated with increased mortality across both short- and longer-term follow-up after proximal femur fracture.

Receiver operating characteristic analysis demonstrated that all three mortality scores showed moderate and largely comparable discriminatory performance across all evaluated timepoints (Table 5). AUC values were stable over time, ranging from 0.723 to 0.729 for NHFS, 0.709 to 0.731 for AHFS, and 0.729 to 0.746 for ACS-NSQIP.

Although ACS-NSQIP consistently yielded numerically higher AUCs from 3 months onward, these differences were small and did not translate into statistically significant superiority. Pairwise comparisons using the DeLong test with Holm correction demonstrated largely comparable discrimination between models across timepoints. Although some comparisons between AHFS and ACS-NSQIP reached statistical significance after correction, the absolute differences in discrimination remained small (Table 6).

Taken together, these findings indicate that all three scores provide similar ability to distinguish between survivors and non-survivors, with only marginal numerical advantages for ACS-NSQIP.

Calibration performance differed more substantially between models but showed consistent patterns across time, with ACS-NSQIP demonstrating closer agreement between predicted and observed mortality (Table 7). The ACS-NSQIP model demonstrated the best overall calibration, with intercepts closest to zero (−0.05 to −0.09) and slopes closest to 1 (0.90–0.94), indicating good agreement between predicted and observed mortality. In addition, ACS-NSQIP consistently achieved the lowest Brier scores at all timepoints (range 0.049–0.176), reflecting superior overall predictive accuracy.

In contrast, both NHFS and AHFS exhibited systematic miscalibration. Intercepts were consistently negative (NHFS: −0.28 to −0.36; AHFS: −0.35 to −0.43), indicating a tendency to overestimate mortality risk. Calibration slopes below 1 (NHFS: 0.77–0.82; AHFS: 0.73–0.79) further suggest overfitting and inadequate spread of predicted risks. Among the models, AHFS showed the poorest calibration, with the most pronounced deviations across all metrics.

Across all models, Brier scores increased over time, reflecting the expected decline in predictive accuracy with longer follow-up. Nevertheless, ACS-NSQIP consistently outperformed NHFS and AHFS in terms of calibration at every timepoint (Table 7).

## 4. Discussion

The observed association between cognitive impairment and mortality likely reflects a complex interaction between cognition, frailty, dependency, comorbidity burden, and reduced physiological reserve.

Mortality in cognitively impaired patients was substantially higher as early as 30 days and continued to diverge over time, reaching more than double that of patients without impairment at 2 years. Both MCI and dementia conferred excess risk over 2 years, independent of age, comorbidity burden, and baseline functional status. This finding is consistent with previous work identifying cognitive dysfunction as a key determinant of outcome in hip fracture populations [8,9], and extends it by showing that even mild impairment carries clinically relevant risk. These findings suggest that cognitive impairment represents an important clinical marker associated with outcome in this population.

However, most existing work has focused on short-term mortality, often limited to 30-day or 1-year endpoints. By extending follow-up to 2 years, the present study provides evidence that the impact of cognitive impairment persists well beyond the immediate postoperative period. This longer-term perspective is particularly relevant, as it captures the cumulative effects of frailty, reduced physiological reserve, and impaired recovery potential, which are not adequately reflected in short-term analyses. Differences in mortality associations across follow-up periods were observed, although these findings should be interpreted cautiously given the absence of statistically significant survival differences between MCI and dementia in unadjusted Kaplan–Meier analysis. Nevertheless, the presence of cognitive impairment overall was consistently associated with increased mortality throughout follow-up. Although mortality increased numerically with severity, survival did not differ significantly between MCI and dementia. This suggests that the presence of any cognitive impairment may mark a threshold of vulnerability, while differences between severity levels emerge more clearly over time and after accounting for confounders. The multivariable analyses support a graded association, with increasing risk across impairment categories.

These findings may support further investigation into whether cognitively impaired patients could benefit from tailored perioperative management, rehabilitation strategies, and follow-up pathways. However, given the retrospective observational design, the present study cannot determine whether such interventions would improve outcomes [10,11]. Patients with MCI, on the other hand, may require sustained support beyond discharge, including structured rehabilitation and closer follow-up to prevent long-term decline [12,13]. These findings may indicate that cognitively impaired patients represent a subgroup requiring closer perioperative attention and structured postoperative follow-up. While patients with dementia appear particularly vulnerable during the early postoperative phase, patients with MCI may remain at increased risk over longer-term follow-up.

Differentiating between degrees of cognitive impairment may help guide allocation of resources and tailor care pathways.

The evaluation of mortality prediction models showed moderate discriminatory performance and largely comparable discrimination across all timepoints. This is in line with previous studies reporting similar performance of hip fracture risk scores [14,15]. However, calibration differed, with ACS-NSQIP demonstrating better agreement between predicted and observed outcomes, while NHFS and AHFS tended to overestimate risk [16]. This may reflect differences between the original development cohorts and the present population, particularly regarding age, comorbidity burden, and prevalence of cognitive impairment [17]. These findings highlight a critical limitation of relying on discrimination alone and underscore the need to consider calibration when applying risk models in clinical practice. The consistent overestimation observed in fracture-specific scores may reflect differences between derivation cohorts and the present population, emphasising the importance of population-specific validation and recalibration.

## 5. Limitations

The retrospective design limits causal interpretation and may introduce residual confounding. Cognitive status was classified retrospectively based on available clinical documentation and cognitive assessments recorded during routine clinical care rather than standardized neuropsychological evaluation. In addition, some potentially relevant frailty-associated and perioperative variables were not systematically available within the dataset. Because excluded cases frequently lacked important baseline information, detailed comparison between included and excluded patients was not consistently feasible.

Cognitive impairment may therefore partly reflect broader vulnerability and baseline health status in this population, including overlap with frailty, sarcopenia, nutritional status and perioperative vulnerability. Finally, the single-center design may limit generalizability to other healthcare settings and populations.

## 6. Conclusions

Cognitive impairment was associated with increased mortality after proximal femur fracture, including in patients with mild cognitive impairment. Although mortality associations varied across follow-up periods, survival differences between mild cognitive impairment and dementia were not statistically significant in unadjusted analyses. The observed associations likely reflect the broader interaction between cognitive impairment, frailty, dependency, and comorbidity burden.

Current mortality prediction tools demonstrated moderate performance in this population. Because excluded cases frequently lacked substantial baseline information, detailed comparative analysis between included and excluded patients was not consistently feasible. Further prospective studies incorporating standardized cognitive assessment and frailty-related variables are required to better define the relationship between cognitive impairment and postoperative outcomes after proximal femur fracture.

## Figures and Tables

**Table 1:** Baseline characteristics of the study cohort stratified by cognitive impairment.

Characteristic	Total	No Impairment	Impairment		*p*-Values
	(*n* = 1579)	(*n* = 1110)	MCI (*n* = 307)	Dementia (*n* = 162)	
**Age (years)**					0.001
Mean ± SD	81.4 ± 9.3	79.9 ± 9.1	82.4 ± 9.0	83.2 ± 9.4	
Median (range)	82 (60–106)	80 (60–104)	83 (61–105)	84 (62–106)	
**Female sex**	1123 (71.1%)	795 (71.6%)	215 (70.0%)	113 (69.8%)	0.79
**BMI (kg/m^2^)**					0.11
Mean ± SD	23.1 ± 4.2	23.4 ± 4.1	22.9 ± 4.3	22.6 ± 4.5	
Median (range)	22.8 (14–38)	23.1 (15–37)	22.6 (14–38)	22.3 (14–36)	
**ASA classification**					0.001
I	49 (3.1%)	40 (3.6%)	7 (2.3%)	2 (1.2%)	
II	585 (37.1%)	478 (43.1%)	85 (27.7%)	22 (13.6%)	
III	845 (53.5%)	540 (48.6%)	190 (61.8%)	115 (70.9%)	
IV	98 (6.2%)	50 (4.5%)	25 (8.2%)	23 (14.3%)	
V	2 (0.1%)	2 (0.2%)	0 (0.0%)	0 (0.0%)	
**Institutionalised**	352 (22.3%)	155 (14.0%)	118 (38.4%)	79 (48.8%)	0.001
**Fracture type**					0.08
Femoral neck (medial)	781 (49.2%)	580 (52.3%)	130 (42.3%)	71 (43.8%)	
Femoral neck (lateral)	17 (1.1%)	12 (1.1%)	3 (1.0%)	2 (1.2%)	
Pertrochanteric	763 (48.3%)	506 (45.6%)	170 (55.4%)	87 (53.7%)	
Subtrochanteric	18 (1.1%)	12 (1.1%)	4 (1.3%)	2 (1.2%)	
**Surgical treatment**					0.07
IM	756 (47.9%)	505 (45.5%)	160 (52.1%)	91 (56.2%)	
HEP	508 (32.2%)	360 (32.4%)	95 (30.9%)	53 (32.7%)	
THA	88 (5.6%)	72 (6.5%)	12 (3.9%)	4 (2.5%)	
DHS	83 (5.3%)	62 (5.6%)	14 (4.6%)	7 (4.3%)	
CS	144 (9.1%)	111 (10.0%)	26 (8.5%)	7 (4.3%)	
**Comorbidities**					
Cardiac disease	304 (19.3%)	180 (16.2%)	78 (25.4%)	46 (28.4%)	0.001
COPD	241 (15.3%)	132 (11.9%)	66 (21.5%)	43 (26.5%)	0.001
Renal disease	158 (10.0%)	74 (6.7%)	48 (15.6%)	36 (22.2%)	0.001
Diabetes	336 (21.3%)	212 (19.1%)	78 (25.4%)	46 (28.4%)	0.06
previous stroke	159 (10.1%)	88 (7.9%)	40 (13.1%)	31 (19.1%)	0.001
Parkinson disease		23 (2.1%)	12 (3.9%	11 (6.8%)	0.018

Data are presented as mean ± standard deviation, median (range), or number (percentage). BMI = body mass index; ASA = American Society of Anesthesiologists; COPD = chronic obstructive pulmonary disease; IM = intramedullary nailing; HEP = hemiarthroplasty; THA = total hip arthroplasty; DHS = dynamic hip screw; CS = cannulated screw.

**Table 2:** Cumulative mortality stratified by cognitive impairment.

Timepoint	No Impairment (*n* = 1110)	MCI (*n* = 307)	Dementia (*n* = 162)
**30 days**	28/1110 (2.52%) 95% CI 1.73–3.63	34/307 (11.07%) 95% CI 7.99–15.10	23/162 (14.20%) 95% CI 9.58–20.53
**3 months**	82/1110 (7.39%) 95% CI 5.95–9.13	58/307 (18.89%) 95% CI 14.89–23.56	36/162 (22.22%) 95% CI 16.37–29.32
**6 months**	108/1110 (9.73%) 95% CI 8.08–11.66	84/307 (27.36%) 95% CI 22.56–32.72	49/162 (30.25%) 95% CI 23.59–37.93
**1 year**	138/1110 (12.43%) 95% CI 10.62–14.49	106/307 (34.53%) 95% CI 29.45–39.95	55/162 (33.95%) 95% CI 27.02–41.64
**18 months**	218/1110 (19.64%) 95% CI 17.42–22.04	118/307 (38.44%) 95% CI 33.12–43.99	82/162 (50.62%) 95% CI 43.04–58.18
**2 years**	266/1110 (23.96%) 95% CI 21.56–26.54	134/307 (43.65%) 95% CI 38.20–49.26	101/162 (62.35%) 95% CI 54.62–69.47

Values represent cumulative mortality. Confidence intervals were calculated using the Wilson method.

**Table 3:** Univariable and multivariable Cox regression analysis for 2-year mortality.

Variable	Univariable HR (95% CI)	*p*-Value	Multivariable HR (95% CI)	*p*-Value
**Cognitive impairment**				
MCI vs. no impairment	2.12 (1.63–2.75)	<0.001	1.48 (1.06–2.08)	0.022
Dementia vs. no impairment	2.63 (2.16–3.19)	<0.001	1.95 (1.54–2.49)	<0.001
**Covariates (multivariable model)**				
Age (per year)	—	—	1.04 (1.03–1.06)	<0.001
Male sex	—	—	2.22 (1.80–2.75)	<0.001
ASA III	—	—	1.73 (1.34–2.23)	<0.001
ASA IV	—	—	2.44 (1.53–3.87)	<0.001
Institutional residence	—	—	1.32 (1.03–1.69)	0.027

Multivariable model adjusted for age, sex, ASA class, institutional residence, and fracture type.

**Table 4:** Logistic regression analysis of mortality at predefined timepoints.

Timepoint	Model	MCI OR (95% CI)	*p*-Value	Holm *p*	Dementia OR (95% CI)	*p*-Value	Holm *p*
30 days	Univariable	1.36 (0.57–3.22)	0.482	0.482	3.54 (1.71–7.34)	<0.001	0.002
	Multivariable	1.20 (0.45–3.24)	0.708	0.708	2.06 (0.77–5.53)	0.150	0.316
3 months	Univariable	2.66 (1.48–4.76)	0.001	0.002	3.58 (2.16–5.92)	<0.001	<0.001
	Multivariable	1.79 (0.89–3.60)	0.105	0.316	2.57 (1.41–4.69)	0.002	0.010
6 months	Univariable	2.93 (1.67–5.13)	<0.001	<0.001	3.38 (2.11–5.40)	<0.001	<0.001
	Multivariable	2.03 (1.07–3.83)	0.032	0.127	2.64 (1.46–4.77)	0.001	0.007
1 year	Univariable	3.09 (1.91–4.98)	<0.001	<0.001	3.57 (2.37–5.37)	<0.001	<0.001
	Multivariable	2.33 (1.44–3.79)	<0.001	0.003	3.04 (1.96–4.73)	<0.001	<0.001
18 months	Univariable	2.55 (1.64–3.95)	<0.001	<0.001	4.20 (2.91–6.06)	<0.001	<0.001
	Multivariable	2.41 (1.50–3.87)	<0.001	0.002	4.48 (2.95–6.79)	<0.001	<0.001
2 years	Univariable	2.47 (1.62–3.75)	<0.001	<0.001	5.24 (3.66–7.51)	<0.001	<0.001
	Multivariable	2.42 (1.44–4.05)	<0.001	0.007	4.79 (3.06–7.51)	<0.001	<0.001

Multivariable models were adjusted for age, sex, ASA class, institutional residence, and fracture type. *p*-values were corrected for multiple comparisons using the Holm–Bonferroni method.

**Table 5:** AUCs of mortality scores for mortality prediction.

Timepoint	NHFS AUC (95% CI)	AHFS AUC (95% CI)	ACS-NSQIP AUC (95% CI)
30 days	0.723 (0.663–0.771)	0.731 (0.682–0.782)	0.729 (0.676–0.780)
3 months	0.723 (0.681–0.755)	0.714 (0.673–0.750)	0.743 (0.702–0.778)
6 months	0.725 (0.691–0.757)	0.709 (0.676–0.742)	0.735 (0.698–0.765)
1 year	0.729 (0.696–0.756)	0.710 (0.676–0.745)	0.741 (0.711–0.772)
18 months	0.727 (0.698–0.755)	0.709 (0.677–0.743)	0.743 (0.712–0.770)
2 years	0.729 (0.700–0.758)	0.712 (0.680–0.745)	0.746 (0.718–0.772)

Area under the receiver operating characteristic curve (AUC) with 95% confidence intervals for the Nottingham Hip Fracture Score (NHFS), Almelo Hip Fracture Score (AHFS), and ACS National Surgical Quality Improvement Program (ACS-NSQIP) model at predefined timepoints. Higher AUC values indicate better discrimination between survivors and non-survivors.

**Table 6:** Pairwise DeLong comparisons of mortality scores with Holm correction.

Timepoint	Comparison	DeLong *p*	Holm-Adjusted *p*
30 days	NHFS vs. AHFS	0.773	1.000
	NHFS vs. ACS-NSQIP	0.875	1.000
	AHFS vs. ACS-NSQIP	0.944	1.000
3 months	NHFS vs. AHFS	0.584	0.805
	NHFS vs. ACS-NSQIP	0.403	0.805
	AHFS vs. ACS-NSQIP	0.107	0.321
6 months	NHFS vs. AHFS	0.287	0.574
	NHFS vs. ACS-NSQIP	0.626	0.626
	AHFS vs. ACS-NSQIP	0.089	0.267
1 year	NHFS vs. AHFS	0.157	0.314
	NHFS vs. ACS-NSQIP	0.419	0.419
	AHFS vs. ACS-NSQIP	0.047	0.141
18 months	NHFS vs. AHFS	0.137	0.275
	NHFS vs. ACS-NSQIP	0.320	0.320
	AHFS vs. ACS-NSQIP	0.042	0.125
2 years	NHFS vs. AHFS	0.127	0.254
	NHFS vs. ACS-NSQIP	0.225	0.225
	AHFS vs. ACS-NSQIP	0.032	0.096

Pairwise comparisons of AUCs between mortality prediction models at each timepoint using the DeLong test. *p*-values were adjusted for multiple comparisons using the Holm–Bonferroni method. Statistical significance indicates differences in discriminatory performance between models.

**Table 7:** Calibration performance of mortality prediction models across all timepoints.

Timepoint	Model	Intercept	Slope	Brier Score
**30 days**	NHFS	−0.28	0.82	0.051
	AHFS	−0.35	0.79	0.053
	ACS-NSQIP	−0.05	0.94	0.049
**3 months**	NHFS	−0.31	0.80	0.097
	AHFS	−0.38	0.77	0.101
	ACS-NSQIP	−0.07	0.92	0.094
**6 months**	NHFS	−0.34	0.79	0.129
	AHFS	−0.41	0.75	0.134
	ACS-NSQIP	−0.08	0.91	0.125
**1 year**	NHFS	−0.36	0.78	0.163
	AHFS	−0.43	0.74	0.168
	ACS-NSQIP	−0.09	0.90	0.158
**18 months**	NHFS	−0.35	0.77	0.175
	AHFS	−0.42	0.73	0.180
	ACS-NSQIP	−0.08	0.91	0.170
**2 years**	NHFS	−0.32	0.78	0.182
	AHFS	−0.41	0.74	0.186
	ACS-NSQIP	−0.08	0.92	0.176

## Data Availability

The data that support the findings of this study are available on request from the corresponding author, D.P. The data are not publicly available due to restrictions containing information that could compromise the privacy of research participants.
